# Clinicopathological significance of SMAD4 loss in pancreatic ductal adenocarcinomas: a systematic review and meta-analysis

**DOI:** 10.18632/oncotarget.14335

**Published:** 2016-12-28

**Authors:** Jin-Dao Wang, Ketao Jin, Xiao-Ying Chen, Jie-Qing Lv, Ke-Wei Ji

**Affiliations:** ^1^ Department of Gastrointestinal Surgery, Shaoxing People's Hospital, Shaoxing Hospital of Zhejiang University, Shaoxing City, Zhejiang Province, China; ^2^ Psychosomatic Second Division, Shaoxing 7th People's Hospital, Shaoxing City, Zhejiang Province, China

**Keywords:** PDAC, SMAD4, DPC4, diagnosis, prognosis

## Abstract

Pancreatic ductal adenocarcinoma (PDAC) is one of the leading causes of cancer mortality. Although advances have been made in understanding the pathogenesis of PDAC, the outcome still remains poor. The aim of this study is to conduct a meta-analysis to evaluate the precise association between SMAD4 loss and clinicopathological significance in PDAC. A literature search was made in PubMed, Web of Science, Google scholar, and EMBASE for related publications. The data were extracted and assessed by two reviewers independently. Analysis of pooled data was performed, Odds Ratio or Hazard Ratio with corresponding confidence intervals was calculated and summarized. 12 relevant articles were included for full review in detail and meta-analysis. The frequency of SMAD4 protein loss was significantly increased in PDAC than in nonmalignant pancreatic tissue, Odd Ratio was 0.05 with 95% confidence interval 0.01-0.23, p<0.0001. SMAD4 loss was significantly associated with poor overall survival in patients with PDAC, Hazard Ratio was 0.61 with 95% confidence interval 0.38-0.99, p=0.05. SMAD4 loss was not correlated with the size, grades, and lymph node metastasis of PDAC. In conclusion, SMAD4 is a biomarker for the diagnosis of PDAC. SMAD4 loss is significantly related to poor prognosis in patients with PDAC.

## INTRODUCTION

Pancreatic ductal adenocarcinoma (PDAC) is one of the leading causes of cancer mortality. Although improvement in clinical management has been made last two decades, the prognosis of PDAC remains poor, and a 5-year survival rate is approximately 5% [1–3]. Because of a lack of specific symptoms and appropriate markers for early stages, most PDAC patients are diagnosed at advanced stages, when radical pancreatic resection is not possible. Therefore, it is critical to identify biomarkers for early diagnosis and development of gene targeted therapy. SMAD4 was also known as the deleted in pancreatic carcinoma 4 (DPC4), is located on chromosome 18q21 [4–5]. SMAD4 is a co-factor that facilitates gene transcription and tumor suppression through the TGF-beta signaling pathway. TGF-beta/*SMAD4* signaling pathway regulates tumor development through mediating growth arrest and inducing apoptosis [5–11]. Previous studies have attempted to correlate the alteration of SMAD4 with clinical features and prognosis in patients with PDAC [12–15]. However, a clear correlation has not been established. We conducted a meta-analysis to investigate the association of SMAD4 status with clinicopathlogical significance and prognosis in patients with PDAC.

## RESULTS

**Figure 1:**
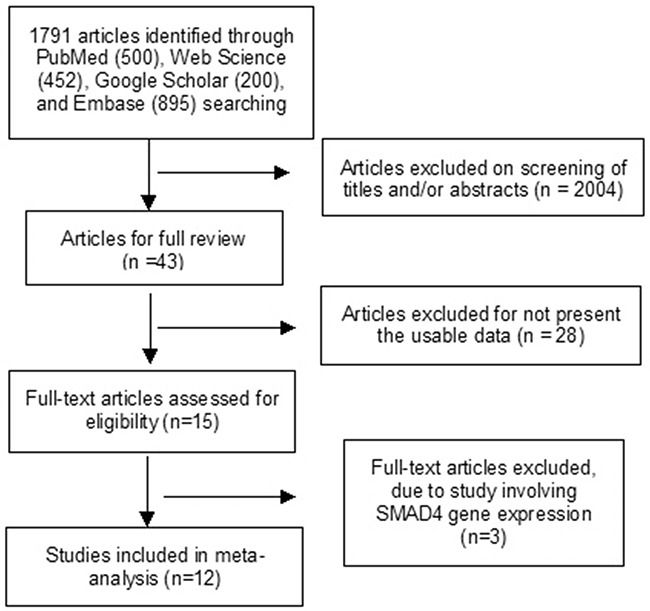
Schematic flow diagram for selection of included studies

12 studies were included for meta-analysis (Figure 1). The main characteristics were listed in Table 1.

**Table 1:** Main Characteristics of included studies

Author	Year	Country	Sample	Patient age	Grade (L/H)	Size (<30/>30)	LN status (-/+)	Follow-up(median)	IHC staining	SMAD4 Ab
Bachet [13]	2012	France	471	Median 63	335/51	225/161	25/291	26MO	nuclear	Santa Cruz 1:100
Biankin [38]	2002	Australia	348	Median 67	38/13		27/24	3.5MO	cytoplasma	Santa Cruz
Javle [39]	2014	USA	91	Mean 60.6	40/20				nuclear	Proteintech Group, Inc.1:450
Handra-Luca [12]	2013	France	99	Median age, Women 61, Men 63.5	62/29	34/57	15/76	26MO	nuclear	Santa Cruz 1:100
Hua [19]	2003	China	34	Median 55	27/7		20/14		nuclear and /or cytoplasma	Santa Cruz 1:100
Oshima [29]	2013	Japan	106	Median 69.5	91/15		34/72		nuclear and /or cytoplasma	Santa Cruz 1:100
Ottenhof [40]	2012	Netherlands	78	Mean 63	61/16		23/54	27MO	nuclear	Santa Cruz 1:300
Tang [41]	2002	China	25							
Tascilar [28]	2001	USA	249	Mean 65.4±10.5		168/141	93/156	17MO	nuclear and /or cytoplasma	Santa Cruz 1:100
Toga [42]	2004	Japan	88	Mean 65.9±9.5	82/6		10/78		nuclear	Santa Cruz 1:100
Xiang [43]	2016	China	241	Median 62			112/129	18.5MO		Abcam 1:150
Zhang [44]	2006	China	30		24/6					

L: Low, H: High; LN: Lymph Node; Ab:antibody; MO: month; IHC: Immunohistochemistry

**Figure 2:**
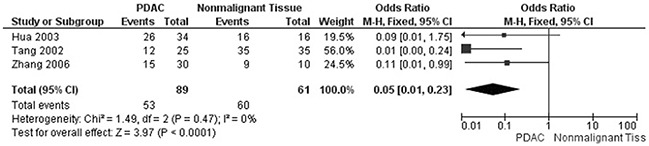
Forest plot for SMAD4 protein expression in PDAC and nonmalignant pancreatic tissue

**Figure 3:**
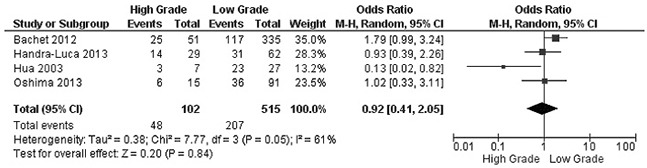
Forest plot for SMAD4 protein expression in different grade of PDAC

**Figure 4:**
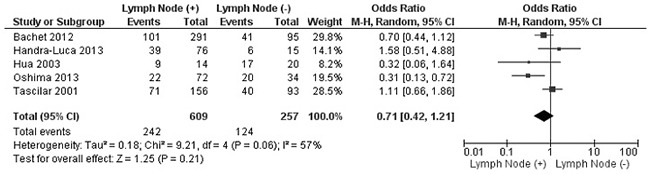
Forest plot for SMAD4 protein expression in different lymph node metastasis status

**Figure 5:**
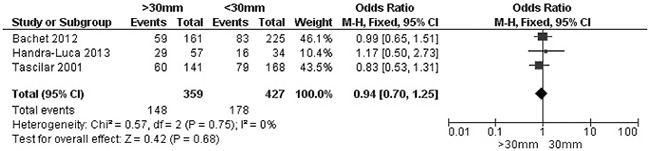
Forest plot for SMAD4 protein expression in different size of PDAC

**Figure 6:**
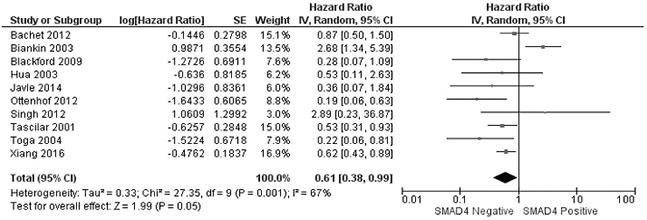
Forest plot for the association of SMAD4 protein expression with overall survival of patients with PDAC

The rate of SMAD4 protein loss was significantly higher in PDCA than in nonmalignant pancreatic tissue (including normal pancreas and hyperplasia), OR was 0.05 with 95% CI 0.01-0.23, z=3.97, p<0.0001, *I*^2^=0% (Figure 2). The frequency of SMAD4 protein loss was similar between high and low grade of PDCA, OR was 0.92 with 95% CI 0.41-2.05, z=0.20, p=0.84, *I*^2^=61% (Figure 3). Loss of SMAD4 protein was not associated with lymph node metastasis status, OR was 0.71 with 95% CI 0.42-1.21, z=1.25, p=0.21, *I*^2^=57% (Figure 4). There was no significantly difference of SMAD4 protein loss rate between large and small size of PDCA tumor, OR was 0.94 with 95% CI 0.70-1.25, z=0.42, p=0.68, *I*^2^=0% (Figure 5). Loss of SMAD4 protein was significantly correlated to overall survival in patients with PDCA, HR was 0.61 with 95% CI 0.38-0.99, z=1.99, p=0.05, *I*^2^=67% (Figure 6).

**Figure 7:**
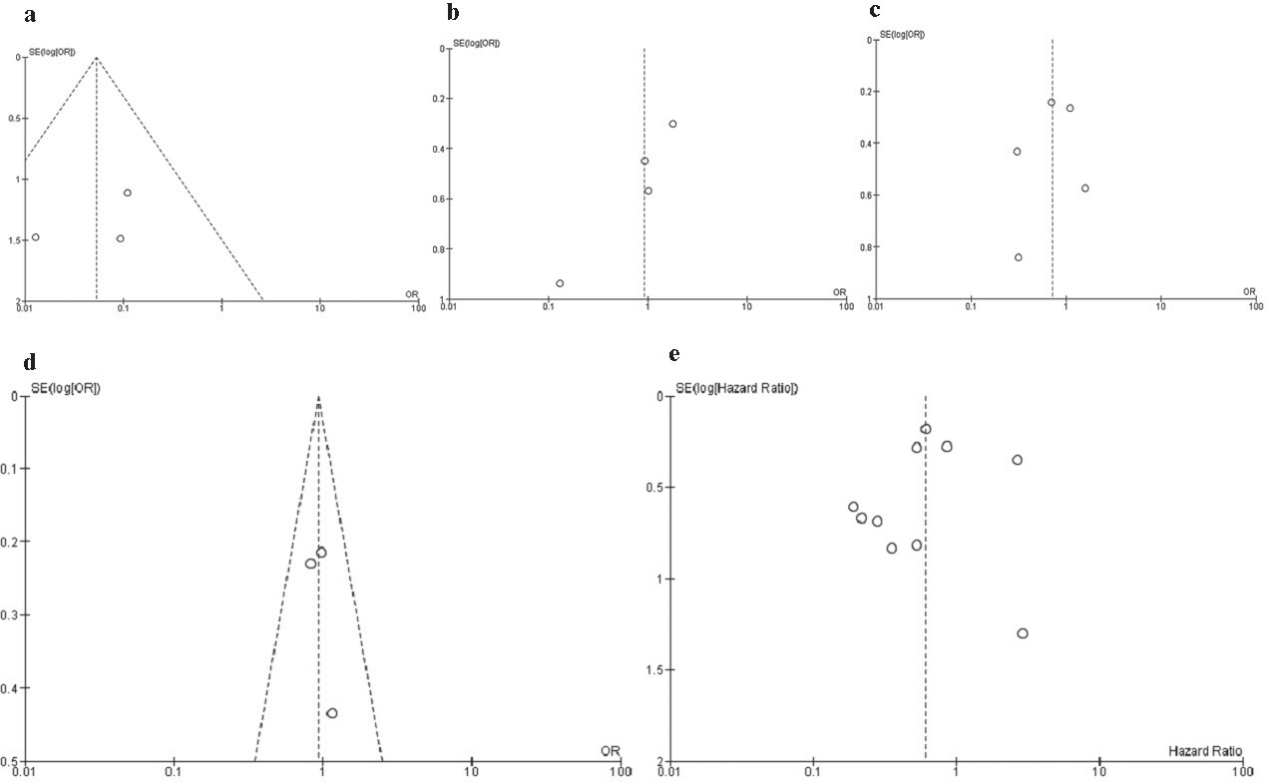
Funnel plot for publication bias Each circle represents the weight of individual study. In X axes, Log(OR)=natural logarithm of OR, Log(HR)=natural logarithm of HR, In Y axes, SE=standard error. **a**. SMAD4 protein expression in PDAC and nonmalignant pancreatic tissue; **b**. SMAD4 protein expression in different grade of PDAC; **c**. SMAD4 protein expression in different lymph node metastasis status; **d**. SMAD4 protein expression in different size of PDAC; **e**. the association of SMAD4 protein expression with overall survival of patients with PDAC.

The methodological quality of each study was assessed separately and independently by JW and XC using Newcastle Ottawa Quality Assessment Scale (NOQAS). This scale for non-randomized case controlled studies and cohort studies was used to allocate a maximum of nine points for the quality of selection, comparability, exposure, and outcomes for study participants. Among 12 studies, two scored 6, six scored 7, and four scored 8. Therefore, the studies were relatively high quality (Table 2). A sensitivity analysis, in which one study was removed at a time, was conducted to assess the result stability. The pooled ORs and HR were not significantly changed, suggesting the stability of our analyses. The funnel plots showed largely symmetric (Figure 7) which indicated there was no publication biases in the meta-analysis of SMAD4 protein expression and clinicopathological features.

**Table 2:** Quality assessment according to the Newcastle–Ottawa scale of the included studies

Author	Selection	Comparability	Exposure	Total score
Bachet [13]	3	2	3	8
Biankin [38]	3	2	3	8
Javle [39]	3	2	2	7
Handra-Luca [12]	2	2	3	7
Hua [19]	3	2	2	7
Oshima [29]	3	2	2	7
Ottenhof [40]	3	2	3	8
Tang [41]	2	2	2	6
Tascilar [28]	3	2	3	8
Toga [42]	2	2	3	7
Xiang [43]	2	2	3	7
Zhang [44]	2	2	2	6

## DISCUSSION

Although tremendous progress has been made last two decades in understanding the pathogenesis of PDAC, five-year survival rates are still less than 5% [7]. The development and progression of PDAC involve various gene alterations including oncogene activation and loss of tumor suppressor gene function [16–18]. The understanding of molecular biology in PDAC contributes to the development of new approaches to its prevention, diagnosis and treatment. Previous studies investigated the relationship of loss of SMAD4 with the features of PDAC and its prognosis in patients, however, the correlation was not clear due to small power of the samples [12–13, 19].

Our data showed the loss of SMAD4 protein expression was significantly increased in PDAC than nonmalignant pancreatic tissues. TGF-beta/*SMAD4* signaling regulates tumor development because of its effects on growth arrest and induced apoptosis [5, 8–9]. Mitogenic growth signals, which are regulated by TGF-beta/*SMAD4*, are required during the process of cell cycle [8, 20]. Therefore, SMAD4 functions as a tumor-suppressor through growth arrest during tumorgenesis. Previous study indicated that TGF-beta could affect growth arrest through upregulating p21 in a SMAD4-dependent manner in colon cancer [21]. Additionally, TIEG, a Zinc-finger encoding gene regulated by TGF-beta/*SMAD*4 signaling was reported to induce apoptosis in pancreatic cells (PCs) [22]. Moreover, *SMAD4* downregulation caused TGF-beta-induced cell cycle arrest and apoptosis, and the restoration of *SMAD4* by gene therapy reversed the invasive phenotype as well as reduced the proliferation in PC cell lines [23–25]. An increase in G1 phase fraction was observed in a PDAC cell lines after inducing SMAD4 expression through a tetracycline system construct. The frequency of SMAD4 protein loss was similar between different sizes as well as different grades of PDAC. More studies are needed to confirm the correlation between SMAD4 status and PDAC features. A number of studies have reported the association between SMAD4 protein loss and lymphatic metastases [13, 19, 26–27], however, the results were inconsistent [27–29]. Pooled OR suggested that the frequency of SMAD4 protein loss was not associated with lymphatic metastasis. TGF-beta/*SMAD4*-independent signaling pathway is activated due to *SMAD4* loss [30]. TGF-beta activates the PI3K/Akt/mTOR pathway and leads to increased migratory capacity and invasiveness during Epithelial-mesenchymal transition (EMT) which facilitates tumor progression and metastasis in PDAC. TGF-beta also suppresses *PTEN* through NF-kappa B and enhanced cell motility and invasiveness [31]. Another activated downstream of TGF-beta/SMAD4-independent signaling is STAT3 which has been proven to be an important regulator for PADC growth, invasion and angiogenesis [32–35]. On the other hand, SMAD4 serves as a tumor-suppressor and mediates cell cycle arrest as well as induced apoptosis in a SMAD4-dependent TGF-beta signaling pathway. The converse role of SMAD4 could be the reason that loss of SMAD4 is not associated with lymph node metastasis in PDAC. Recently Hingorani and colleagues used genetically engineered mouse model and found RUNX3 expression together with the *DPC4/SMAD4* haploinsufficiency could inform metastasis status in PDAC [36]. More studies need to be finished in future for clinical application.

Although substantial progress has been made in understanding the pathogenesis of PDAC over last two decades, the prognosis is still poor. Multiple studies have been conducted showing inconsistencies of the association between SMAD4 expression status and prognosis in PDAC patients due to small samples [19, 29, 37–38]. In present study, the pooled data from ten studies indicated SMAD4 loss is significantly associated with prognosis in patients with PDAC. Finally our study selected all the published articles written in English and Chinese, did not include some relevant articles written in other languages or unpublished papers which may result in certain publication bias. Therefore, the result should be interpreted carefully.

In conclusion, SMAD4 is a biomarker for the diagnosis of PDAC. SMAD4 loss is significantly related with poor prognosis of patients with PDAC. SMAD4 loss is not associated with lymph node metastasis of PDAC.

## MATERIALS AND METHODS

The meta-analysis was performed by using PRISMA checklist as a guide (Supplementary Checklist S1).

### Selection criteria and study search

Systematic review of several databases was conducted in July 2016 with no lower limit set for date of publication. Following electronic databases were searched for relevant articles without any language restrictions: PubMed, Web of Science, Google scholar, and EMBASE. The keywords “SMAD4” or “DPC4” and “pancreatic cancer” or “pancreatic ductal adenocarcinoma” or pancreatic adenocarcinoma” were used for relative articles searching. There were 500 articles identified from PubMed, 452 articles from Web Science, 895 articles from EMBASE. 15,800 articles were identified from Google Scholar, first 200 of them were screened since the rest of them were not related to present study. A total of 2047 articles were initially identified by the search strategy, and 43 full-text articles were retrieved after screening. Forward and backward citation chasing of each selected article was performed in case they included another study of interest that had not been indentified. Studies were selected based on the following criteria: 1) The association between SMAD4 protein expression and the clinicopathological features of PDAC; 2) The association of SMAD4 protein expression and prognosis in patients with PDAC. SMAD4 protein expression was examined by immunochemistry. The following exclusion criteria were used: 1) the studies investigated the association between SMAD4 mRNA expression and clinicopathological significance 2) the studies utilized the same population or overlapping database, 3) the studies utilized cell lines or mice.

### Data extraction and study assessment

Two reviewers (JW and XC) extracted data from selected studies independently. Any disagreement was discussed and reached a consensus for all issues. The following items were collected from each study: first author's name, year of publication, geographical location, age of patients, sample size, grades, size of the tumor, lymph node metastasis status, immunohistostaining, SMAD4 antibodies used, and HRs with 95% CIs from multivariate analyses.

### Statistics analysis

Odds ratios (OR) and hazard ratio with their 95% confidence intervals were calculated. Heterogeneity among studies was estimated using the Cochran's Q statistic and *I*^2^ tests. The *I^2^* statistic was used to examine the difference for between study variability due to heterogeneity rather than chance, with a range from 0 to 100 percent. A fixed effect model was used for *I*^2^ <50%, while a random effect model was used for *I*^2^>50%. The analysis was performed to compare the frequency of SMAD4 protein expression between PDCA and nonmalignant prostate tissue. In addition, we evaluated the frequency of SMAD4 protein expression in different grades, different size of the tumor, and the correlation between SMAD4 expression and lymph node metastasis status, as well as the relationship between SMAD4 expression and prognosis in patients with PDCA. All p values were two sided. Funnel plots were used for detection of publication bias. All analysis was performed with Review Manager 5.2 (Cochrane Collaboration, Software Update, Oxford, UK).

## SUPPLEMENTARY CHECKLIST




