# Supplementary material for: Clinicopathological significance of SMAD4 loss in pancreatic ductal adenocarcinomas: a systematic review and meta-analysis
Source: Oncotarget. 2016 Dec 28;8(10):16704–11. doi: 10.18632/oncotarget.14335 (PMC5369995; doi:10.18632/oncotarget.14335)
Supplement: Supplementary file 1 [file oncotarget-08-16704-s001.pdf]

Clinicopathological significance of SMAD4 loss in pancreatic ductal adenocarcinomas: a systematic review and meta-analysis

SUPPLEMENTARY CHECKLIST

Supplementary Checklist 1: PRISMA Checklist.

See Supplementary File 1
